# Identification of differentially expressed genes in non-small cell lung cancer

**DOI:** 10.18632/aging.102521

**Published:** 2019-12-09

**Authors:** Ke Wang, Ruo Chen, Zhuan Feng, Yu-Meng Zhu, Xiu-Xuan Sun, Wan Huang, Zhi-Nan Chen

**Affiliations:** 1 National Translational Science Center for Molecular Medicine and Department of Cell Biology, Fourth Military Medical University, Shaanxi 710032, China

**Keywords:** NSCLC, bioinformatics, biomarkers, TOP2A

## Abstract

Lung cancer is the most common malignant tumor and the leading cause of cancer-related deaths worldwide. Because current treatments for advanced non-small cell lung cancer (NSCLC), the most prevalent lung cancer histological subtype, show limited efficacy, screening for tumor-associated biomarkers using bioinformatics reflects the hope to improve early diagnosis and prognosis assessment. In our study, a Gene Expression Omnibus dataset was analyzed to identify genes with prognostic significance in NSCLC. Upon comparison with matched normal tissues, 118 differentially expressed genes (DEGs) were identified in NSCLC, and their functions were explored through bioinformatics analyses. The most significantly upregulated DEGs were TOP2A, SLC2A1, TPX2, and ASPM, all of which were significantly associated with poor overall survival (OS). Further analysis revealed that TOP2A had prognostic significance in early-stage lung cancer patients, and its expression correlated with levels of immune cell infiltration, especially dendritic cells (DCs). Our study provides a dataset of potentially prognostic NSCLC biomarkers, and highlights TOP2A as a valuable survival biomarker to improve prediction of prognosis in NSCLC.

## INTRODUCTION

Lung cancer is the most common tumor worldwide, and carries the highest morbidity and mortality rates [1]. Lung cancer is classified into two major histological subtypes, small cell lung cancer (SCLC; 13% of cases) and non-small cell lung cancer (NSCLC; 83% of cases). Surgical resection is seldom an option for SCLC treatment, owing to typical advanced-stage diagnosis; thus, most SCLC patients receive chemotherapy, but its efficacy is generally limited. On the other hand, only a small number of early-stage NSCLC patients can be treated with surgery, which achieves a 5-year survival rate as high as 70% in patients with stage IA NSCLC [2]. Chemotherapy or radiotherapy are also indicated in patients with more advanced NSCLC, but are associated with a 5-year survival rate of only ~23%. While some success is being achieved with newer immunological and targeted therapies for NSCLC, there are still significant limitations precluding their use in many cases [3]. Notwithstanding, the low 5-year survival rate for patients with lung cancer is largely due to insufficient preventive efforts and generalized late diagnosis [4].

Bioinformatics analysis allows screening of tumor-associated biomarkers from large data repositories to assist early diagnosis and prognostic assessment of cancer [5, 6]. For example, mining of publicly available genomic repositories (i.e. The Gene Expression Omnibus database (GEO) and The Cancer Genome Atlas (TCGA) database) led to identification of a subset of cancer-dysregulated miRNAs, which may allow early detection of pre-cancerous and cancerous oral lesions [7], and of tumor microenvironment-related genes that predict poor outcomes in glioblastoma patients [8].

In our study, a GEO dataset was selected for identification of differentially expressed genes (DEGs) in NSCLC. Gene ontology (GO), Kyoto Encyclopedia of Genes and Genomes (KEGG), and protein-protein interaction (PPI) network analyses were used to link DEGs’ genomic and functional information. In addition, data retrieved from TCGA and GTEx projects was evaluated through Gene Expression Profiling Interactive Analysis (GEPIA) to further assess the presence of relevant DEGs in NSCLC subtypes. Among the most significant DEGs, the TOP2A gene encoding human topoisomerase IIα (TOPIIα) emerged as a potential prognostic biomarker for early-stage lung cancer. Furthermore, its expression was negatively correlated with tumor infiltration of immune cells (especially dendritic cells, DCs) in NSCLC samples. While functional studies are needed to complement our findings, the biomarker dataset provided by our study may serve to improve early diagnosis of NSCLC and help advance new therapeutic strategies.

## RESULTS

### Identification of DEGs in NSCLC

**Figure 1:**
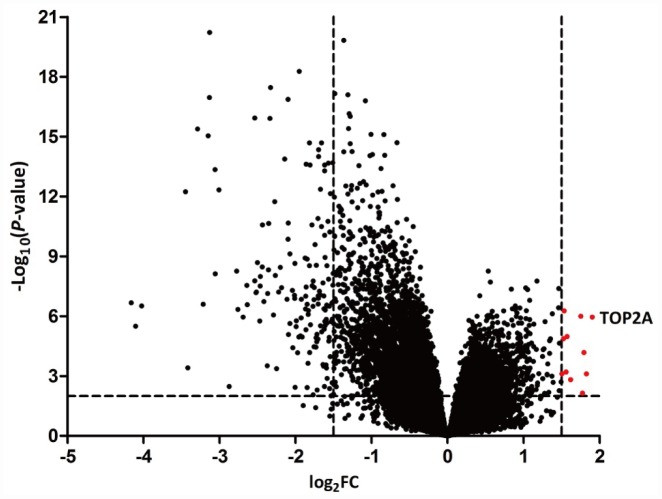
**Volcano plots of DEGs in NSCLC.** NSCLC samples were analyzed against matched normal lung tissues in the GEO GSE103512 dataset. Data points in red represent upregulated genes. TOP2A was the most significant DEG.

The GEO dataset GSE103512 was selected for identification of DEGs in 60 human NSCLC specimens against 9 matched normal tissue samples using the GEO2R tool. Genes were defined as DEGs if they had a log_2_FC > 1.5 or < -1.5 and *p* < 0.01. A total of 118 genes were identified as DEGs; among these, 11 were upregulated and 107 were downregulated in NSCLC (Figure 1). A full DEG list is shown in Supplementary Table 1.

### GO enrichment analysis of DEGs

**Figure 2:**
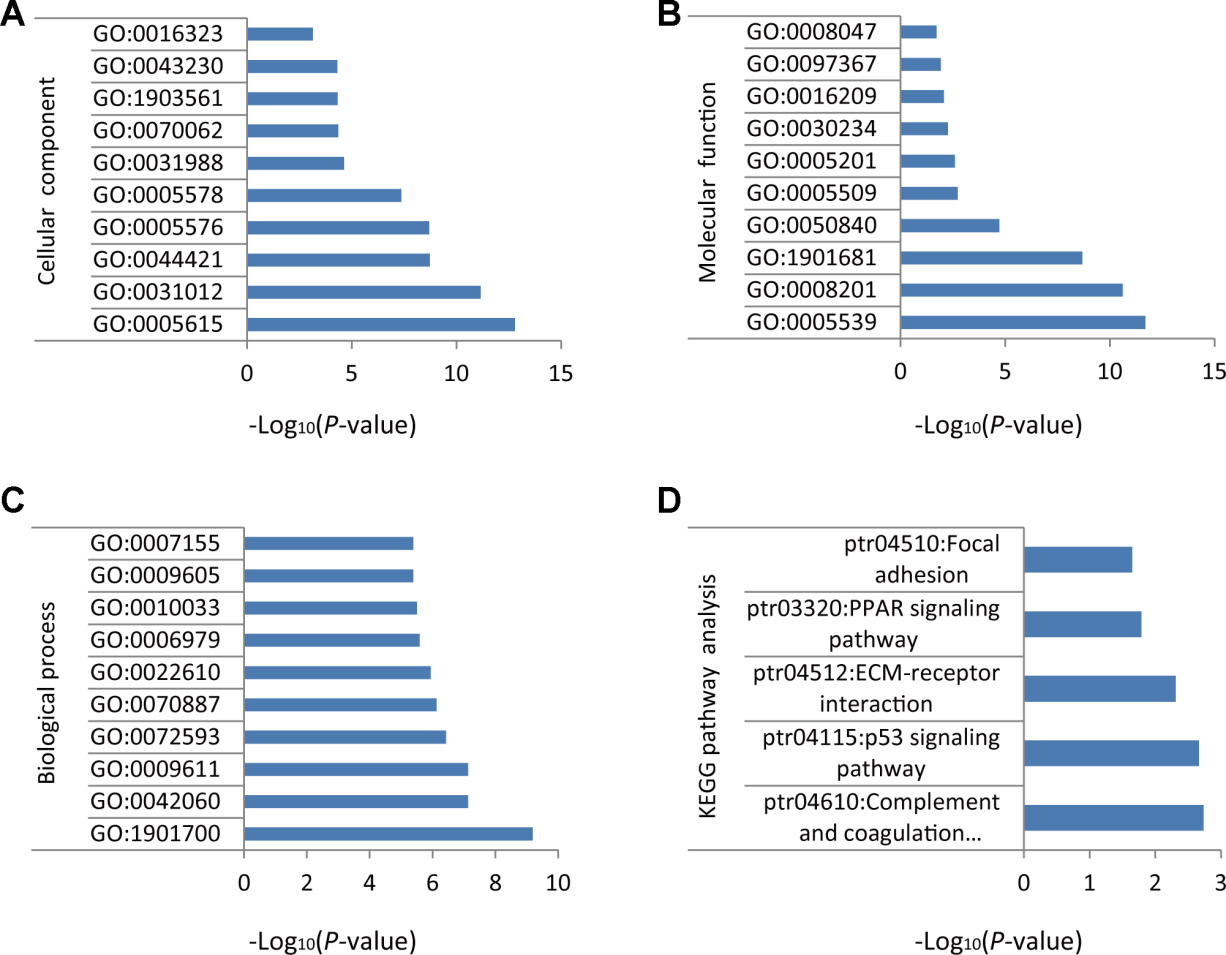
**GO and KEGG enrichment analysis of DEGs in NSCLC.** (**A**) Cellular component. (**B**) Molecular function. (**C**) Biological process. (**D**) Biochemical and signal transduction pathways revealed by KEGG pathway analysis.

GO enrichment analysis was conducted on the 118 DEGs identified above. For cellular component (CC), the top ten terms were GO: 0005615~extracellular space, GO: 0031012~extracellular matrix, GO: 0044421~extracellular region part, GO: 0005576~extracellular region, GO: 0005578~proteinaceous extracellular matrix, GO: 0031988~membrane-bounded vesicle, GO: 0070062~ extracellular exosome, GO: 1903561~extracellular vesicle, GO: 0043230~extracellular organelle, and GO: 0016323~basolateral plasma membrane (Figure 2A). For molecular function (MF), the top ten terms were GO: 0005539~glycosaminoglycan binding, GO: 0008201~heparin binding, GO: 1901681~sulfur compound binding, GO: 0050840~extracellular matrix binding, GO: 0005509~calcium ion binding, GO: 0005201~extracellular matrix structural constituent, GO: 0030234~enzyme regulator activity, GO: 0016209~antioxidant activity, GO: 0097367~carbohydrate derivative binding, and GO: 0008047~enzyme activator activity (Figure 2B). For biological process (BP), the top ten terms were GO: 1901700~response to oxygen-containing compound, GO: 0042060~wound healing, GO: 0009611~response to wounding, GO: 0072593~reactive oxygen species metabolic process, GO: 0070887~cellular response to chemical stimulus, GO: 0022610~biological adhesion, GO: 0006979~response to oxidative stress, GO: 0010033~response to organic substance, GO: 0009605~response to external stimulus, and GO: 0007155~cell adhesion (Figure 2C).

### KEGG pathway analysis of DEGs

KEGG pathway analysis was performed using the Database for Annotation, Visualization and Integrated Discovery (DAVID) v6.8. Results indicated that the DEGs identified in NSCLC samples were mainly related to ‘complement and coagulation cascades’, ‘p53 signaling pathway’, ‘ECM-receptor interaction’, ‘PPAR signaling pathway’, and ‘focal adhesion’ (Figure 2D).

### Validation of upregulated DEGs

**Figure 3:**
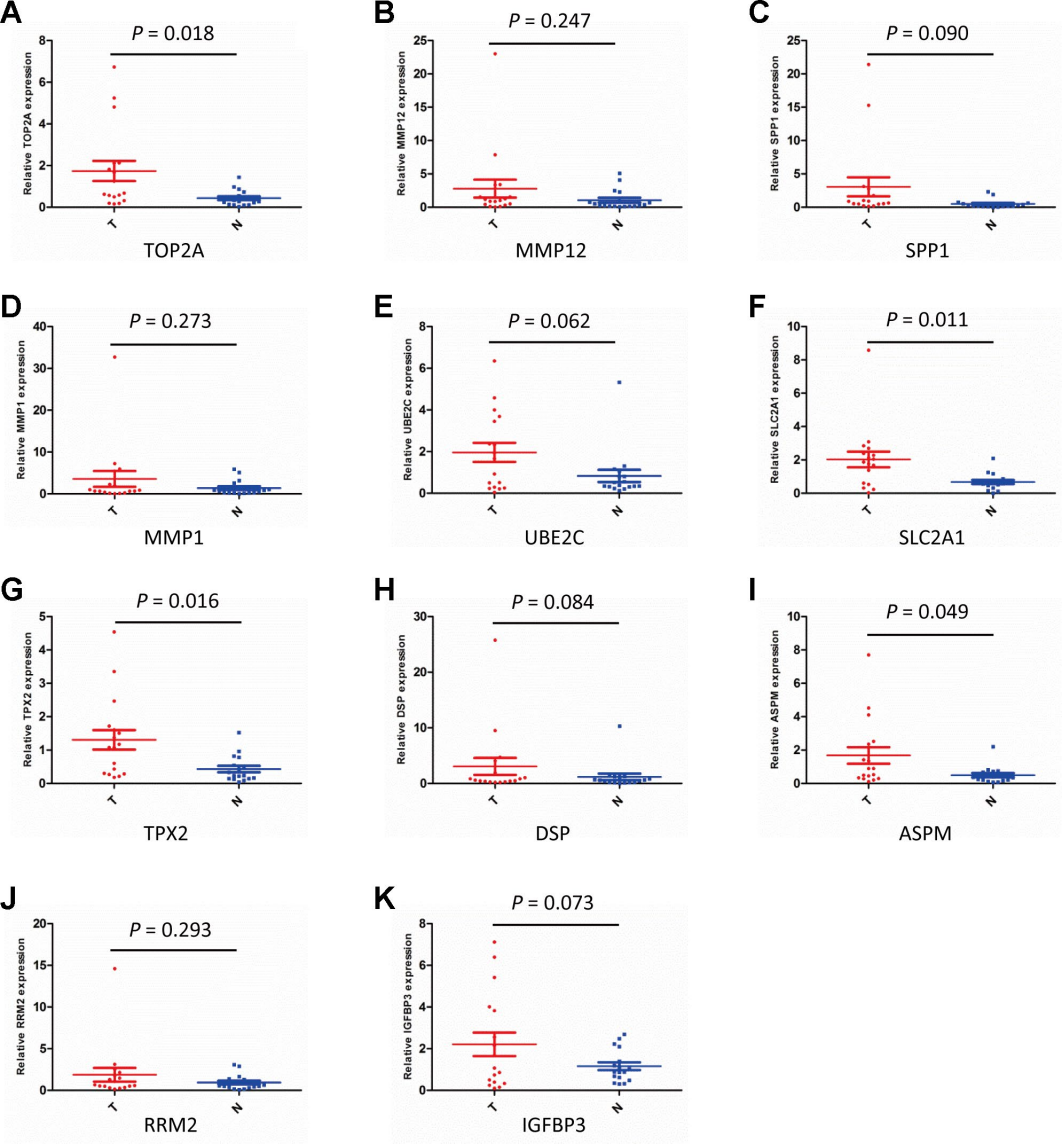
**Validation of DEGs expression by qPCR.** (**A**–**K**) Detection of DEGs expression in NSCLC (T) and adjacent non-tumor lung specimens (**N**) using qPCR. (n = 17; *P* < 0.05 indicates significance).

The DEGs upregulated in NSCLC were selected for validation by quantitative real-time PCR (qPCR) on 17 paired NSCLC/adjacent non-tumor samples collected from surgical patients. The overall trend indicated that all the upregulated DEGs from the GEO database were also overexpressed at the mRNA level in our clinical NSCLC specimens. However, overexpression in NSCLC samples vs normal lung tissues was only significant for TOP2A (*P* = 0.018), SLC2A1 (*P* = 0.011), TPX2 (*P* = 0.016), and ASPM (*P* = 0.049) (Figure 3A–3K).

**Figure 4:**
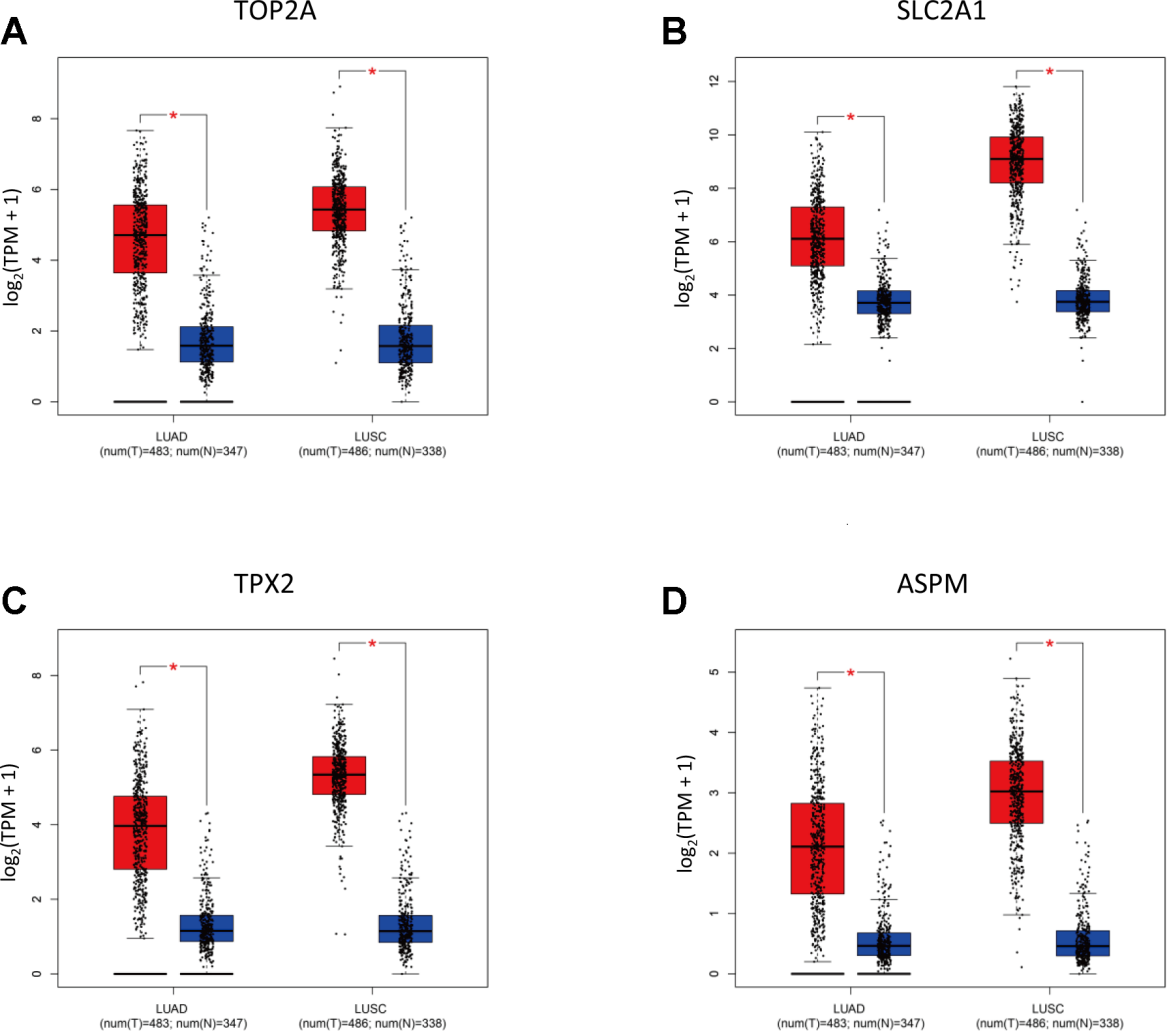
**Validation of selected DEGs by GEPIA.** (**A**–**D**) Expression of TOP2A, SLC2A1, TPX2, and ASPM in NSCLC subtypes (LUAD, n = 483; LUSC, n = 486) and normal lung tissues.

Using Gene Expression Profiling Interactive Analysis (GEPIA), a newly developed interactive web server for analyzing RNA-Seq expression data, we confirmed on NSCLC datasets retrieved from TCGA and GTEx projects that TOP2A (*P* < 0.05), SLC2A1 (*P* < 0.05), TPX2 (*P* < 0.05), and ASPM (*P* < 0.05) were upregulated in lung adenocarcinoma (LUAD) and lung squamous cell carcinoma (LUSC) specimens, compared to adjacent normal lung samples (Figure 4A–4D).

### Protein-protein interaction network and correlation analysis of upregulated DEGs

**Figure 5:**
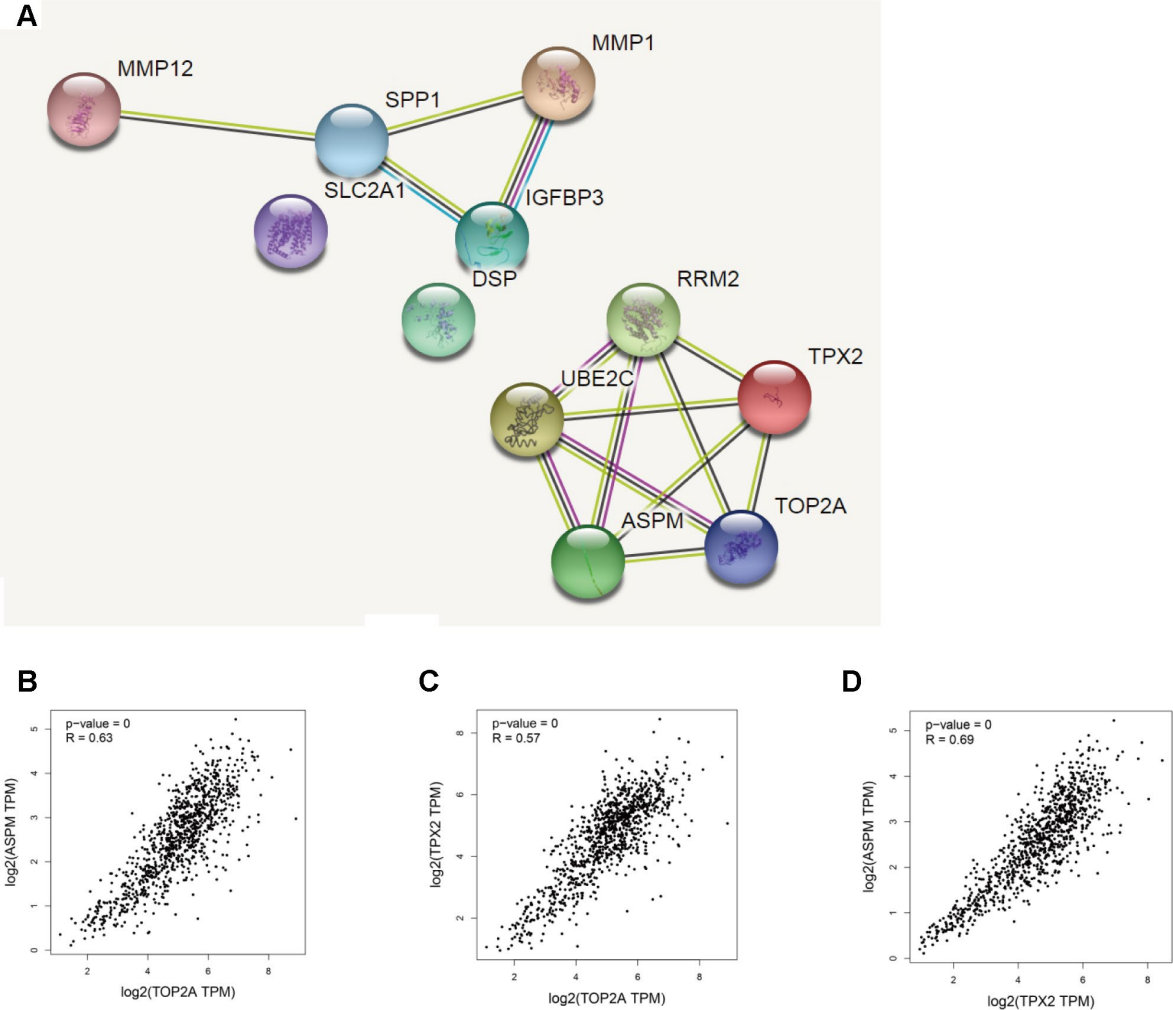
**Correlation analysis of DEGs in NSCLC.** (**A**) PPI network of upregulated DEGs. (**B**–**D**) Analysis of TOP2A, TPX2, and ASPM by GEPIA’s correlation model. The correlation coefficients for TOP2A & ASPM, TOP2A & TPX2, and TPX2 & ASPM were 0.63, 0.57, and 0.69, respectively (*P* = 0.000).

We used the STRING database (https://string-db.org/) to construct protein-protein interaction (PPI) networks for 11 DEGs upregulated in NSCLC (Figure 5A). Results showed that TOP2A, TPX2, and ASPM were interconnected. GEPIA was next used to conduct correlation analysis on these three genes. The correlation coefficients for TOP2A & ASPM, TOP2A & TPX2, and TPX2 & ASPM were 0.63, 0.57, and 0.69 respectively (*P* = 0.000) (Figure 5B–5D). These data suggest that overexpression of TOP2A, TPX2, and ASPM may significantly impact the development or progression of NSCLC.

### Correlations between upregulated DEGs in NSCLC and patient survival

**Figure 6:**
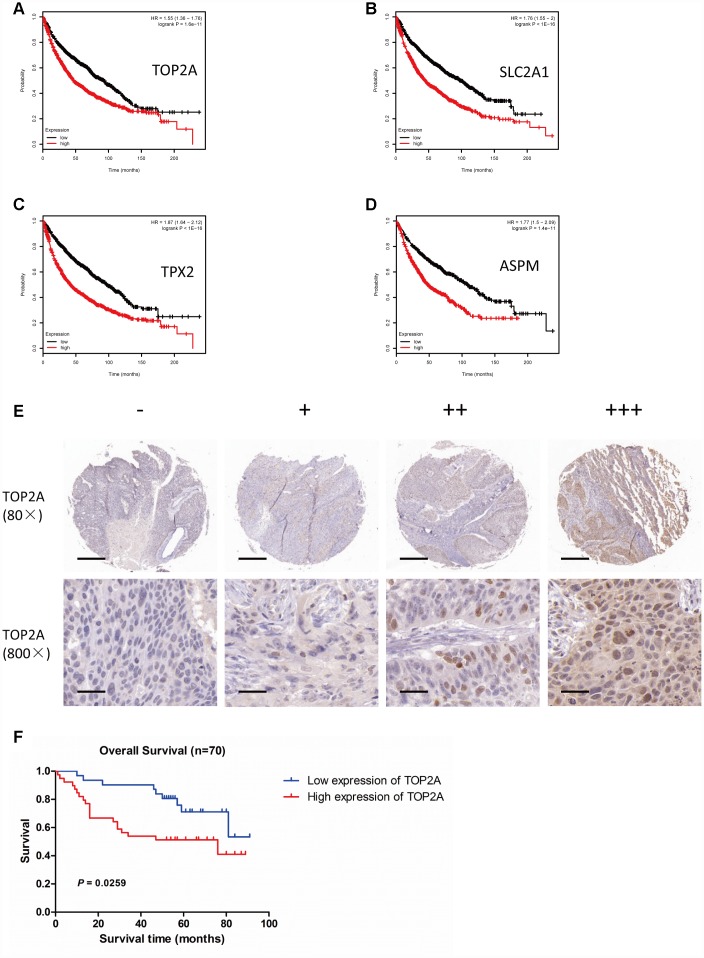
**Association of TOP2A, SLC2A1, TPX2, and ASPM with overall survival in patients with lung cancer. Survival analyses were conducted using the Kaplan Meier plotter tool.** (**A**) OS based on high vs low TOP2A expression (*P* = 1.6e-11). (**B**) OS based on high vs low SLC2A1 expression (*P* < 1e-16). (**C**) OS based on high vs low TPX2 expression (*P* < 1e-16). (**D**) OS based on high vs low ASPM expression (*P* = 1.4e-11). (**E**) IHC analysis of TOP2A expression in NSCLC (80X and 800X magnifications). (**F**) OS of NSCLC patients with high or low TOP2A protein expression (*P* = 0.0259).

To assess whether upregulation of TOP2A, SLC2A1, TPX2, and ASPM in NSCLC is correlated with patient overall survival (OS), we interrogated NSCLC datasets using the Kaplan Meier plotter platform (http://kmplot.com/analysis/). Results showed that high expression of TOP2A, SLC2A1, TPX2, or ASPM was significantly associated with poor OS (*P* = 1.6e-11, *P* < 1e-16, *P* < 1e-16, and *P* = 1.4e-11, respectively) (Figure 6A–6D).

To confirm the relationship between TOP2A (the most significantly upregulated DEG) and patient prognosis, we analyzed TOP2A protein expression in a tissue microarray (TMA) of NSCLC samples by immunohistochemistry (IHC). TOP2A signal localized mainly in the nucleus and to a lesser extent in the cytoplasm of tumor cells. TOP2A immunoreactivity was next categorized into four levels, i.e. “-”, “+”, “++”, and “+++”, according to the intensity and density of TOP2A-positive cells in each sample (Figure 6E). Using sample-associated clinical data, Kaplan-Meier analysis confirmed that patients with high TOP2A expression had significantly worse OS (*P* = 0.0259) (Figure 6F). These results strongly suggest that assessment of TOP2A expression, at the protein and/or mRNA levels, could be a valuable aid for NSCLC prognosis evaluation.

To further investigate the possible impact of TOP2A expression on lung cancer, we analyzed the relationship between TOP2A expression and clinical characteristics of lung cancer patients in the Kaplan-Meier plotter databases (Table 1). High TOP2A expression was significantly associated with poor OS in both female (*P* = 1.3e-05) and male (*P* = 5.8e-06) patients. In addition, high TOP2A expression was associated with poor OS in Stage 1 (*P* = 9.6e-08), Stage T1 (*P* = 7.6e-05), Stage N0 (*P* = 3.6e-04), and Stage M0 (*P* = 3.2e-05) patients. These results indicated that TOP2A expression levels can inform prognosis in early-stage lung cancer patients. Therefore, we propose that TOP2A may serve as an efficient survival biomarker to significantly improve the prediction of NSCLC prognosis.

**Table 1:** Correlation of TOP2A mRNA expression and clinical prognosis in lung cancer for different clinicopathological factors.

**Clinicopathological characteristic**	**Overall survival (n = 1926)**		
	**N**	**Hazard ratio**	***P***
**Gender**			
**Female**	715	1.68(1.33-2.13)	1.3E-05
**Male**	1100	1.44(1.23-1.69)	5.8E-06
**Smoking**			
**Yes**	820	1.37(1.11-1.68)	0.0031
**No**	205	1.38(0.79-2.41)	0.26
**Grade**			
**I**	201	1.09(0.76-1.56)	0.63
**II**	310	1.23(0.9-1.69)	0.19
**III**	77	1.24(0.64-2.4)	0.52
**Stage**			
**1**	577	2.14(1.61-2.85)	9.6E-08
**2**	244	0.97(0.67-1.4)	0.88
**3**	70	0.96(0.56-1.66)	0.89
**4**	4	－	－
**AJCC Stage T**			
**1**	437	1.78(1.33-2.39)	7.6E-05
**2**	589	1.3(1.04-1.63)	0.02
**3**	81	1.35(0.82-2.24)	0.24
**4**	46	1.02(0.54-1.91)	0.95
**AJCC Stage N**			
**0**	781	1.47(1.19-1.81)	3.6E-04
**1**	252	1.34(0.98-1.84)	0.064
**2**	111	0.96(0.64-1.44)	0.84
**AJCC Stage M**			
**0**	681	1.55(1.26-1.91)	3.2E-05
**1**	10	－	－

OS analyses were conducted using the Kaplan-Meier plotter online platform.

### TOP2A expression correlates with immune infiltration in NSCLC

**Figure 7:**
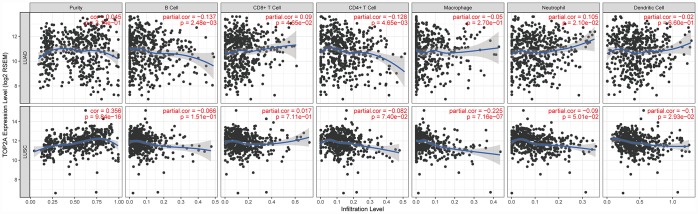
**Correlation of TOP2A expression with immune cell infiltration levels in LUAD and LUSC.** Tumor-infiltrating immune cells included B cells, CD4+ T cells, CD8+ T cells, neutrophils, macrophages, and DCs. Gene expression levels against tumor purity are displayed in the left-most panel.

**Figure 8:**
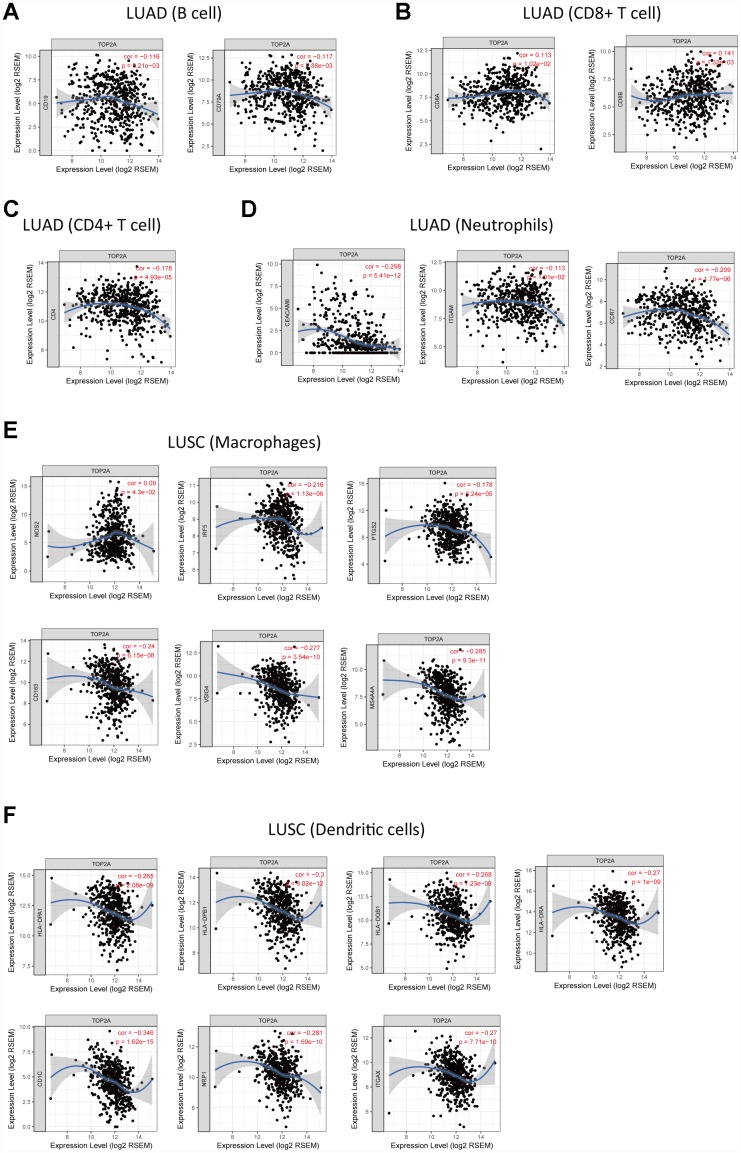
**Correlation of TOP2A expression with gene markers of tumor-infiltrating immune cells in NSCLC.** (**A**) Correlation with gene markers of B cells in LUAD. (**B**) Correlation with gene markers of CD8+ T cells in LUAD. (**C**) Correlation with gene markers of CD4+ T cells in LUAD. (**D**) Correlation with gene markers of neutrophils in LUAD. (**E**) Correlation with gene markers of macrophages in LUSC. (**F**) Correlation with gene markers of DCs in LUSC.

Tumor-infiltrating immune cells may restrict or promote tumor growth and thus play a critical role in tumor development. Therefore, we investigated the relationship between TOP2A expression and immune infiltration in NSCLC using Tumor IMmune Estimation Resource (TIMER), which allows systematic analysis of immune infiltrates across diverse cancer types (https://cistrome.shinyapps.io/timer/). Six immune cell types (B cells, CD4+ T cells, CD8+ T cells, neutrophils, macrophages, and DCs) were assessed by TIMER against TCGA lung cancer datasets. Results showed a slight correlation of TOP2A expression with B cells (partial.cor = -0.137, *P* = 2.48e-03), CD4+ T cells (partial.cor = -0.128, *P* = 4.65e-03), CD8+ T cells (partial.cor = 0.09, *P* = 4.65e-02), and neutrophils (partial.cor = 0.105, *P* = 2.10e-02) in LUAD, and with macrophages (partial.cor = -0.225, *P* = 7.16e-07) and DCs (partial.cor = -0.1, *P* = 2.93e-02) in LUSC (Figure 7). The further define the correlation between TOP2A expression and immune infiltrates in lung cancer, gene markers of tumor-infiltrating immune cells were also evaluated. Again, slight correlations with TOP2A levels were determined for gene markers of B cells, CD4+ T cells, CD8+ T cells, neutrophils, macrophages, and DCs (Figure 8A–8F). Especially, we detected a more significant negative correlation between TOP2A expression and gene markers of DCs in LUSC (HLA-DPA1: cor = -0.265, *P* = 2.08e-09; HLA-DPB1: cor = -0.3, *P* = 9.02e-12; HLA-DQB1: cor = -0.268, *P* = 1.23e-09; HLA-DRA: cor = -0.27, *P* = 1e-09; CD1C: cor = -0.346, *P* = 1.62e-15; NRP1: cor = -0.281, *P* = 1.69e-10; ITGAX: cor = -0.27, *P* = 7.71e-10) (Figure 8F). Therefore, we speculate that TOP2A overexpression might influence antitumor immune responses in the NSCLC microenvironment.

## DISCUSSION

Among all malignant tumors, lung cancer currently carries the highest incidence (11.6%) and mortality rate (18.4%) in the world. [9]. Although it is widely recognized that new cancer cases could be avoided by eliminating or reducing exposure to known lifestyle and environmental risk factors [10], the current burden of lung cancer requires urgent efforts to identify key genes with diagnostic and prognostic significance.

Our study interrogated 60 NSCLC samples and 9 matched normal lung controls in a GEO dataset to identify DEGs in NSCLC. A total of 118 genes (11 upregulated and 107 downregulated) were identified as DEGs. GO analysis showed that most of these DEGs were related to structures or functional processes affecting the extracellular space/matrix, suggestive of diverse roles in the tumor microenvironment. Association with GO ‘glycosaminoglycan binding’ molecular function, as well as GO ‘oxygen-containing compound’ and ‘wound healing’ biological processes indicated the DEGs’ involvement in the anti-inflammatory response. These findings were supported by KEGG pathway analysis, which showed significant correlations with the complement system.

In general, the genes that were highly expressed in tumor tissues had known tumor-promoting effects, while the lowly expressed genes generally mediate tumor-suppressing effects. For validation analysis, we focused on putative tumor-promoting genes, which are more directly targetable and have therefore greater potential clinical applicability. Overall, our qPCR assays on 17 independent NSCLC samples confirmed that all the upregulated DEGs in the GEO dataset were also expressed at higher levels in our NSCLC samples, compared to matched non-tumor controls. However, mRNA overexpression in our NSCLC cohort was only significant for TOP2A, SLC2A1, TPX2, and ASPM. We speculate that increasing sampling size may still lead to validation of more DEGs. In addition, GEPIA analysis demonstrated that TOP2A, SLC2A1, TPX2, and ASPM were upregulated in clinical samples from two major subtypes of NSCLC, i.e. LUAD and LUSC, which stresses the potential relevance of these DEGs in NSCLC development and/or progression. Moreover, these findings are partially supported by a previous gene expression profiling study that identified TOP2A and TPX2 as putative biomarkers of NSCLC [11].

The TOP2A gene is located at 17q12-21 and encodes the human topoisomerase IIα (TOPIIα), which mediates DNA decatenation by rejoining DNA double strand breaks to separate entangled sister chromatides during cell division [12, 13]. TOP2A is highly expressed in dividing cells, and is considered as a proliferation marker in both normal and tumor cells. TOP2A is highly expressed in esophageal, liver, gastric, breast, and colorectal cancers. In breast cancer, high expression of TOP2A is associated with low expression of estrogen receptor (ER) and high expression of Ki-67, and was proposed to be an important prognostic molecular indicator [12, 14–18]. The SLC2A1 gene encodes GLUT1, a glucose transporter that mediates a rate-limiting step for glucose metabolism in cancer cells [19–21]. SLC2A1 is considered an early marker of malignant tumors, overexpressed in esophageal squamous cell carcinoma, gastric carcinoma, and colon cancer, among others, often in association with poor prognosis [22–25]. The TPX2 gene is located at 20q11.2 and encodes a microtubule-associated protein involved in spindle assembly during cell mitosis. TPX2 overexpression is common to many tumor types. In hepatocellular carcinoma, it was correlated with increased proliferation, apoptosis inhibition, and induction of EMT [26]. In breast cancer, TPX2 silencing repressed PI3K/AKT and activated p53 signaling, which inhibited proliferation and promoted apoptosis [27]. The ASPM gene, located at 1q31, encodes a 3477 amino-acid-long protein involved in mitotic spindle regulation and DNA double-strand break repair. ASPM overexpression has been associated with the development of various tumors [28, 29]. In hepatocellular carcinoma, ASPM was suggested to be a novel marker for vascular invasion, early recurrence, and poor prognosis [28]. In prostate cancer, high ASPM expression correlated with tumor progression and predicted poor outcome [29]. Altogether, the above findings from diverse tumor types are consistent with our expression data and our PPI network results, suggesting that TOP2A, TPX2, and ASPM function interconnectedly to increase mitotic rate in tumor cells. In subsequent studies, TOP2A-, TPX2-, and ASPM-specific knockout cell and animal models could be used to validate the contribution of each gene to NSCLC progression and survival.

Our survival analyses on the Kaplan-Meir plotter tool indicated that upregulation of TOP2A, SLC2A1, TPX2, and ASPM independently predicted poor OS in NSCLC patients. Moreover, for TOP2A, high expression was associated with poor OS in Stage 1, Stage T1, Stage N0, and Stage M0 NSCLC patients. An association between TOP2A and poor OS was further confirmed by assessing protein expression by IHC in a NSCLC TMA. These results indicated that TOP2A expression levels may aid prognosis evaluation in early-stage lung cancer patients.

The role of TOP2A in development/progression of NSCLC is still unclear. Since our GO and KEGG enrichment analyses indicated that the identified DEGs were also involved in immune responses, we assessed molecular markers of tumor-infiltrating immune cells, which critically affect early anti-tumor responses and often sustain tumor growth through immuno-suppressive actions. TOP2A expression was slightly correlated with B cells, CD4+ T cells, CD8+ T cells, and neutrophils in LUAD, and with macrophages and DCs in LUSC. In particular, a more significant negative correlation between TOP2A expression and HLA-complex members, CD1C, NRP1, and ITGAX expression in DCs was detected in LUSC. Since DCs are crucial antigen presenting cells (APCs) that trigger T-cell mediated antitumor immunity [30], impaired function of tumor-infiltrating DCs may seriously affect the body's anti-tumor immune response. Although further proof is clearly needed to establish a causal relationship, these data suggest that TOP2A overexpression may impair DC-mediated anti-tumor immune response in NSCLC.

In summary, through bioinformatics analyses we showed that TOP2A, SLC2A1, TPX2, and ASPM are overexpressed in NSCLC and show significant association with poor OS. As cell-cycle dependent proteins with interrelated functions, TOP2A, TPX2, and ASPM play key roles in the mitotic machinery that drives tumor cell replication in NSCLC and other tumor types. Further analysis confirmed that TOP2A expression was correlated with the prognosis of early-stage lung cancer patients and was negatively correlated with immune cell infiltration in NSCLC, especially of DCs. Thus, our study provided a potential biomarker dataset for NSCLC prognosis and suggested that TOP2A, in particular, may be a valuable survival biomarker to improve prognostic efforts and possibly guide new therapeutic developments for NSCLC.

## MATERIALS AND METHODS

### GEO dataset

The GEO dataset GSE103512 [31] was selected for our study. The platform for GSE103512 is GPL13158, [HT_HG-U133_Plus_PM] Affymetrix HT HG-U133+ PM Array, which includes 280 formalin-fixed, paraffin embedded normal and tumor samples of four cancer types (breast, colorectal, prostate, and non-small cell lung cancer). The array contains 65 breast cancer samples with 10 matched normal samples; 57 colorectal cancer samples with 12 matched normal samples; 60 NSCLC samples with 9 matched normal samples; and 60 prostate cancer samples with 7 matched normal samples. We only analyzed NSCLC samples and their matched controls for DEG identification.

### Identification of DEGs

Gene expression analysis of NSCLC and matched normal tissues was performed with the GEO2R tool (https://www.ncbi.nlm.nih.gov/geo/geo2r/?acc=GSE103512). DEGs were sorted by log_2_FC > 1.5 or < -1.5 and *p* < 0.01.

### Enrichment analysis

GO enrichment and KEGG pathway analyses were performed using DAVID v6.8 (https://david.ncifcrf.gov/), an online set of functional annotation tools to infer biological activities for large gene lists [32, 33]. *P* < 0.05 denoted statistical significance.

### RNA extraction and qPCR analysis

For qPCR validation of DEGs defined in the GEO dataset GSE103512, 17 paired NSCLC and adjacent non-tumor lung samples were collected from patients who underwent surgery in the Department of Thoracic Surgery (Tangdu Hospital, Fourth Military Medical University). The study was approved by the Ethics Committee of First Affiliated Hospital of Fourth Military Medical University (KY20183327-1). Total RNA was extracted from samples using Total RNA Kit II (Omega Bio-tek, GA, USA) following manufacturer’s instructions, and then reversely transcribed to cDNA using the PrimeScript RT Reagent Kit (TaKaRa, Kusatsu, Japan). qPCR was carried out using the SYBR Premix Ex Taq II Kit (TaKaRa, Kusatsu, Japan). PCR primers are listed in Table 2.

**Table 2:** Sequences of PCR primers.

**Primer**	**Sequence**
TOP2A-Forward Primer	ACCATTGCAGCCTGTAAATGA
TOP2A-Reverse Primer	GGGCGGAGCAAAATATGTTCC
MMP12-Forward Primer	CATGAACCGTGAGGATGTTGA
MMP12-Reverse Primer	GCATGGGCTAGGATTCCACC
SPP1-Forward Primer	CTCCATTGACTCGAACGACTC
SPP1-Reverse Primer	CAGGTCTGCGAAACTTCTTAGAT
MMP1-Forward Primer	AAAATTACACGCCAGATTTGCC
MMP1-Reverse Primer	GGTGTGACATTACTCCAGAGTTG
UBE2C-Forward Primer	GACCTGAGGTATAAGCTCTCGC
UBE2C-Reverse Primer	TTACCCTGGGTGTCCACGTT
SLC2A1-Forward Primer	GGCCAAGAGTGTGCTAAAGAA
SLC2A1-Reverse Primer	ACAGCGTTGATGCCAGACAG
TPX2-Forward Primer	ATGGAACTGGAGGGCTTTTTC
TPX2-Reverse Primer	TGTTGTCAACTGGTTTCAAAGGT
DSP-Forward Primer	GCAGGATGTACTATTCTCGGC
DSP-Reverse Primer	CCTGGATGGTGTTCTGGTTCT
ASPM-Forward Primer	GGCCCTAGACAACCCTAACGA
ASPM-Reverse Primer	AGCTTGGTGTTTCAGAACATCA
RRM2-Forward Primer	CACGGAGCCGAAAACTAAAGC
RRM2-Reverse Primer	TCTGCCTTCTTATACATCTGCCA
IGFBP3-Forward Primer	AGAGCACAGATACCCAGAACT
IGFBP3-Reverse Primer	GGTGATTCAGTGTGTCTTCCATT
GAPDH-Forward Primer	GCACCGTCAAGGCTGAGAAC
GAPDH-Reverse Primer	TGGTGAAGACGCCAGTGGA

### GEPIA-based analysis of RNA-sequencing expression data

GEPIA [34] is a newly developed interactive web server for analyzing RNA-Seq expression data of 9,736 tumors and 8,587 normal samples from TCGA and GTEx projects using a standard processing pipeline (http://gepia.cancer-pku.cn/index.html). It is developed by Zefang Tang, Chenwei Li, and Boxi Kang (Zhang Lab, Peking University), and provides customizable functions such as differential expression analysis, profiling according to cancer type or pathological stage, patient survival analysis, similar gene detection, and correlation and dimensionality reduction analyses. We selected NSCLC specimens and normal lung tissues for differential expression analysis, and DEGs for correlation analysis. The Spearman method was used to determine significant correlations.

### PPI network analysis

PPI network analysis was performed on DEGs using STRING software (https://string-db.org/) [35].

### Survival analysis

The Kaplan Meier plotter [36, 37] (http://kmplot.com/analysis/) is an open source software that allows to assess the effect of 54,000 genes on survival in 21 cancer types. TOP2A, SLC2A1, TPX2, and ASPM were the DEGs selected for validation as survival biomarkers by the Kaplan Meier plotter. OS was calculated using Kaplan-Meier analysis and log-rank test.

### Immunohistochemistry

A TMA of 90 NSCLC and matched normal samples was purchased from Shanghai Biochip Company (Shanghai, China). Twenty paired samples were excluded from analysis owing to incomplete patient information and/or sample absence. Therefore, IHC was performed as reported previously [4] on 70 matched specimens. Paraffin sections were dewaxed, followed by antigen retrieval with Tris-EDTA buffer (pH 9). Deparaffinized sections were treated with methanol containing 3% hydrogen peroxide for 15 min, washed with PBS, and incubated with blocking serum for 30 min. Then, sections were incubated with anti-TOP2A (66541-1-Ig, Proteintech, USA) diluted 1:100, at 4°C overnight. Immunoperoxidase staining was conducted using a streptavidin-peroxidase kit and 3,3′-diaminobenzidine (Zhongshan Jinqiao Co., Beijing, China). Hematoxylin was used to counterstain the nuclei. Intensity and density of TOP2A-positive cells was evaluated and scored as reported before [4].

### TIMER analysis

TIMER (https://cistrome.shinyapps.io/timer/) [38, 39] is a comprehensive resource for systematic analysis of immune infiltrates across diverse cancer types using RNA-Seq expression profiling data. Six immune cell types (B cells, CD4+ T cells, CD8+ T cells, neutrophils, macrophages, and dendritic cells) were assessed by TIMER on NSCLC sample data, and the correlation between TOP2A expression and immune infiltration was determined. In addition, we assessed the correlations between TOP2A expression and gene markers of tumor-infiltrating immune cells [40].

### Statistical analysis

Data were analyzed using GraphPad Prism 5.0. Expression levels of DEGs between NSCLC and matched normal tissues were compared by paired two-tailed t-test. OS was calculated using Kaplan-Meier analysis and log-rank test. *P* < 0.05 was considered significant.

## Supplementary Material

Supplementary Table 1
