# Supplementary material for: Identification of differentially expressed genes in non-small cell lung cancer
Source: Aging (Albany NY). 2019 Dec 9;11(23):11170–85. doi: 10.18632/aging.102521 (PMC6932904; doi:10.18632/aging.102521)
Supplement: Supplementary Table 1 [file aging-11-102521-s001..docx]

**Supplementary Table 1. GEO Dataset analysis of upregulated and downregulated genes in NSCLC compared to their normal samples.**

| ID | Gene symbol | Log_2_FC (T vs N) | *P*-value |
| --- | --- | --- | --- |
| Up-regulated |  |  |  |
| 201291_PM_s_at | TOP2A | 1.90240004 | 1.10E-06 |
| 204580_PM_at | MMP12 | 1.82506806 | 7.83E-04 |
| 209875_PM_s_at | SPP1 | 1.79415539 | 6.64E-05 |
| 204475_PM_at | MMP1 | 1.77447335 | 7.15E-03 |
| 202954_PM_at | UBE2C | 1.75120012 | 1.01E-06 |
| 201250_PM_s_at | SLC2A1 | 1.617461 | 1.52E-03 |
| 210052_PM_s_at | TPX2 | 1.57310608 | 1.04E-05 |
| 200606_PM_at | DSP | 1.55935977 | 6.25E-04 |
| 239002_PM_at | ASPM | 1.53476134 | 5.37E-07 |
| 209773_PM_s_at | RRM2 | 1.52564644 | 1.34E-05 |
| 212143_PM_s_at | IGFBP3 | 1.50307071 | 7.71E-04 |
| Down-regulated |  |  |  |
| 207194_PM_s_at | ICAM4 | -1.50481551 | 7.14E-09 |
| 222513_PM_s_at | SORBS1 | -1.50737538 | 2.75E-08 |
| 235489_PM_at | RHOJ | -1.51133369 | 2.01E-14 |
| 202995_PM_s_at | FBLN1 | -1.51515639 | 1.40E-06 |
| 210762_PM_s_at | DLC1 | -1.51760949 | 1.65E-06 |
| 212951_PM_at | ADGRF5 | -1.51900517 | 2.18E-03 |
| 207134_PM_x_at | TPSB2///TPSAB1 | -1.52127714 | 2.18E-07 |
| 209101_PM_at | CTGF | -1.52969627 | 1.87E-06 |
| 204437_PM_s_at | FOLR1 | -1.53234874 | 9.84E-03 |
| 223577_PM_x_at | MALAT1 | -1.53968876 | 7.07E-13 |
| 212328_PM_at | LIMCH1 | -1.54429388 | 1.07E-04 |
| 202878_PM_s_at | CD93 | -1.54852253 | 5.91E-11 |
| 202376_PM_at | SERPINA3 | -1.55061541 | 4.28E-03 |
| 205960_PM_at | PDK4 | -1.55483769 | 3.11E-07 |
| 1554966_PM_a_at | FILIP1L | -1.55724731 | 1.16E-05 |
| 201041_PM_s_at | DUSP1 | -1.56266461 | 1.78E-05 |
| 220088_PM_at | C5AR1 | -1.5669677 | 1.40E-08 |
| 233980_PM_s_at | VWF | -1.5670232 | 2.14E-14 |
| 244108_PM_at | SYNPO2 | -1.57378938 | 1.76E-11 |
| 211663_PM_x_at | PTGDS | -1.57785676 | 3.07E-07 |
| 217757_PM_at | A2M | -1.58350337 | 2.15E-06 |
| 219747_PM_at | NDNF | -1.58552964 | 1.31E-03 |
| 222068_PM_s_at | DNAAF1 | -1.58710526 | 2.92E-09 |
| 224358_PM_s_at | MS4A7 | -1.58904743 | 2.78E-07 |
| 1569203_PM_at | CXCL2 | -1.59385971 | 3.98E-04 |
| 203821_PM_at | HBEGF | -1.59681832 | 1.45E-06 |
| 230378_PM_at | SCGB3A1 | -1.59802695 | 2.79E-04 |
| 203065_PM_s_at | CAV1 | -1.60839867 | 2.90E-06 |
| 228434_PM_at | BTNL9 | -1.61343526 | 1.13E-09 |
| 204223_PM_at | PRELP | -1.61405072 | 1.82E-06 |
| 219059_PM_s_at | LYVE1 | -1.6172687 | 5.12E-14 |
| 224061_PM_at | INMT | -1.61734479 | 2.63E-14 |
| 224321_PM_at | TMEFF2 | -1.6187797 | 8.75E-11 |
| 204919_PM_at | PRR4 | -1.62102558 | 3.35E-03 |
| 205569_PM_at | LAMP3 | -1.62648882 | 4.27E-07 |
| 205236_PM_x_at | SOD3 | -1.62941374 | 9.68E-08 |
| 1557924_PM_s_at | ALPL | -1.63256413 | 3.22E-04 |
| 200878_PM_at | EPAS1 | -1.63926668 | 2.15E-08 |
| 202340_PM_x_at | NR4A1 | -1.65637674 | 6.11E-06 |
| 216339_PM_s_at | TNXB///TNXA | -1.65918431 | 3.18E-11 |
| 223395_PM_at | ABI3BP | -1.65998421 | 2.03E-15 |
| 229759_PM_s_at | VEPH1 | -1.67196177 | 3.00E-06 |
| 235666_PM_at | ITGA8 | -1.67290221 | 4.37E-13 |
| 239827_PM_at | RGCC | -1.67474239 | 8.54E-08 |
| 209555_PM_s_at | CD36 | -1.68603649 | 5.58E-08 |
| 209763_PM_at | CHRDL1 | -1.69631761 | 9.77E-15 |
| 213247_PM_at | SVEP1 | -1.69697087 | 4.54E-15 |
| 201531_PM_at | ZFP36 | -1.70327544 | 2.43E-07 |
| 209267_PM_s_at | SLC39A8 | -1.70386723 | 1.40E-05 |
| 202018_PM_s_at | LTF | -1.71694058 | 5.04E-03 |
| 201694_PM_s_at | EGR1 | -1.72267154 | 1.92E-05 |
| 203088_PM_at | FBLN5 | -1.72722947 | 2.78E-09 |
| 209541_PM_at | IGF1 | -1.73068961 | 1.22E-05 |
| 225987_PM_at | STEAP4 | -1.73165152 | 3.51E-05 |
| 209335_PM_at | DCN | -1.74219771 | 2.54E-10 |
| 221132_PM_at | CLDN18 | -1.75520315 | 3.60E-08 |
| 209189_PM_at | FOS | -1.7560903 | 3.41E-05 |
| 201496_PM_x_at | MYH11 | -1.75813383 | 1.50E-07 |
| 219597_PM_s_at | DUOX1 | -1.76860237 | 1.73E-08 |
| 214354_PM_x_at | SFTPB | -1.76909776 | 8.73E-03 |
| 205624_PM_at | CPA3 | -1.77078737 | 3.21E-07 |
| 207574_PM_s_at | GADD45B | -1.78231498 | 2.96E-08 |
| 230670_PM_at | IGSF10 | -1.78317976 | 2.67E-11 |
| 1555745_PM_a_at | LYZ | -1.81592287 | 6.61E-05 |
| 210096_PM_at | CYP4B1 | -1.81846029 | 2.93E-09 |
| 1555809_PM_at | CRISPLD2 | -1.83002185 | 5.11E-07 |
| 203021_PM_at | SLPI | -1.83600157 | 2.30E-04 |
| 206754_PM_s_at | CYP2B7P///CYP2B6 | -1.84663993 | 3.74E-03 |
| 229584_PM_at | LRRK2 | -1.84752612 | 2.52E-07 |
| 213895_PM_at | EMP1 | -1.84832103 | 1.37E-09 |
| 200795_PM_at | SPARCL1 | -1.86161074 | 3.48E-08 |
| 202291_PM_s_at | MGP | -1.86432741 | 4.73E-06 |
| 203305_PM_at | F13A1 | -1.86501592 | 1.28E-09 |
| 210081_PM_at | AGER | -1.87565027 | 1.21E-09 |
| 201427_PM_s_at | SEPP1 | -1.91396477 | 1.11E-05 |
| 227697_PM_at | SOCS3 | -1.92797973 | 1.21E-06 |
| 215775_PM_at | THBS1 | -1.93576384 | 9.64E-06 |
| 201539_PM_s_at | FHL1 | -1.9500333 | 5.35E-19 |
| 201525_PM_at | APOD | -1.95630379 | 1.11E-05 |
| 210068_PM_s_at | AQP4 | -1.98885426 | 1.69E-06 |
| 207542_PM_s_at | AQP1 | -1.99130637 | 3.29E-06 |
| 222486_PM_s_at | ADAMTS1 | -2.00988684 | 2.25E-09 |
| 212670_PM_at | ELN | -2.01290248 | 8.98E-08 |
| 203649_PM_s_at | PLA2G2A | -2.03479268 | 3.85E-05 |
| 209116_PM_x_at | HBB | -2.07337986 | 7.52E-10 |
| 227480_PM_at | SUSD2 | -2.07470608 | 2.33E-06 |
| 223623_PM_at | C2orf40 | -2.0972188 | 2.15E-11 |
| 204894_PM_s_at | AOC3 | -2.09808947 | 1.37E-10 |
| 209074_PM_s_at | FAM107A | -2.10013673 | 1.33E-17 |
| 217414_PM_x_at | HBA2///HBA1 | -2.11256097 | 1.42E-07 |
| 212713_PM_at | MFAP4 | -2.14294123 | 1.32E-14 |
| 227099_PM_s_at | C11orf96 | -2.18366609 | 3.72E-08 |
| 207978_PM_s_at | NR4A3 | -2.20101015 | 3.53E-09 |
| 204213_PM_at | PIGR | -2.2487476 | 4.31E-04 |
| 214199_PM_at | SFTPD | -2.26475605 | 9.04E-09 |
| 203548_PM_s_at | LPL | -2.28918246 | 8.66E-07 |
| 203813_PM_s_at | SLIT3 | -2.33771836 | 1.20E-16 |
| 202992_PM_at | C7 | -2.35312406 | 2.22E-11 |
| 214091_PM_s_at | GPX3 | -2.43647615 | 2.65E-11 |
| 204575_PM_s_at | MMP19 | -2.43862819 | 3.40E-09 |
| 202768_PM_at | FOSB | -2.4651178 | 3.05E-08 |
| 218835_PM_at | SFTPA2 | -2.87230439 | 3.32E-03 |
| 209612_PM_s_at | ADH1B | -3.00714247 | 4.60E-13 |
| 208250_PM_s_at | DMBT1 | -3.05704828 | 7.48E-09 |
| 205392_PM_s_at | CCL15-CCL14///CCL15///CCL14 | -3.05860864 | 4.42E-14 |
| 226304_PM_at | HSPB6 | -3.13439326 | 1.08E-17 |
| 38691_PM_s_at | SFTPC | -3.21631669 | 2.50E-07 |
